# Correction: Estimating impact of food choices on life expectancy: A modeling study

**DOI:** 10.1371/journal.pmed.1003962

**Published:** 2022-03-25

**Authors:** Lars T. Fadnes, Jan-Magnus Økland, Øystein A. Haaland, Kjell Arne Johansson

The following information is missing from the Funding statement: This work was supported by Trond Mohn foundation, grant number TMS2019TMT02. The hosting for the computations were performed on the Norwegian Research and Education Cloud (NREC), using resources provided by the University of Bergen and the University of Oslo (http://www.nrec.no/). The funders had no role in the study design, analysis, decision to publish, or preparation of the manuscript.
