# Supplementary material for: Estimating impact of food choices on life expectancy: A modeling study
Source: PLoS Med. 2022 Feb 8;19(2):e1003889. doi: 10.1371/journal.pmed.1003889 (PMC8824353; doi:10.1371/journal.pmed.1003889)
Supplement: S1 Text — (PDF) [file pmed.1003889.s001.pdf]

S1 Text: Medline/PubMed search to estimate number of nutritional articles per year.

(("Nutritional Sciences"[Mesh] OR "Nutritional Status"[Mesh] OR "Child Nutrition Sciences"[Mesh] OR "Parenteral Nutrition Solutions"[Mesh] OR "Nutritional Physiological Phenomena"[Mesh] OR "Diet, Food, and Nutrition"[Mesh] OR "Enteral Nutrition"[Mesh] OR "Infant Nutrition Disorders"[Mesh] OR "Nutrition Disorders"[Mesh] OR "Nutrition Surveys"[Mesh] OR "Parenteral Nutrition"[Mesh] OR "Parenteral Nutrition, Total"[Mesh] OR "Child Nutrition Disorders"[Mesh] OR "Nutrition Assessment"[Mesh] OR "Parenteral Nutrition, Home"[Mesh] OR "Parenteral Nutrition, Home Total"[Mesh] OR "Animal Nutrition Sciences"[Mesh] OR "Fetal Nutrition Disorders"[Mesh] OR "Nutrition Policy"[Mesh] OR "Nutrition Therapy"[Mesh]) OR ("Elder Nutritional Physiological Phenomena"[Mesh] OR "Sports Nutritional Physiological Phenomena"[Mesh] OR "Sports Nutritional Sciences"[Mesh] OR "Prenatal Nutritional Physiological Phenomena"[Mesh] OR "Nutritive Value"[Mesh] OR "Nutritional Requirements"[Mesh] OR "Animal Nutritional Physiological Phenomena"[Mesh] OR "Maternal Nutritional Physiological Phenomena"[Mesh] OR "Adolescent Nutritional Physiological Phenomena"[Mesh] OR "Infant Nutritional Physiological Phenomena"[Mesh] OR "Child Nutritional Physiological Phenomena"[Mesh] OR "Food Assistance"[Mesh] OR "Food Labeling"[Mesh] OR "Parenteral Nutrition Solutions" [Pharmacological Action])) AND ("2019/01/01"[Date - Publication] : "2019/12/31"[Date - Publication])

- ➔ 2020: Yielded 36,097 results.
    - o Assumed to be impacted by Covid-19 and might not be representative
- ➔ 2019: Yielded 71,131 results.
- ➔ 2018: Yielded 71,140 results.
- ➔ 2017: Yielded 67,918 results.
