# Supplementary material for: Estimating impact of food choices on life expectancy: A modeling study
Source: PLoS Med. 2022 Feb 8;19(2):e1003889. doi: 10.1371/journal.pmed.1003889 (PMC8824353; doi:10.1371/journal.pmed.1003889)
Supplement: S2 Text — (PDF) [file pmed.1003889.s002.pdf]

S2 Text: String used in PubMed to identify meta-analyses for setting hazard ratios.

(grains[title/abstract] OR fruits[title/abstract] OR vegetables[title/abstract] OR nuts[title/abstract] OR legumes[title/abstract] OR fish[title/abstract] OR eggs[title/abstract] OR milk[title/abstract] OR dairy[title/abstract] OR meat*[title/abstract] OR "sugar-sweetened beverages"[title/abstract]) AND mortality[title/abstract] AND Meta-Analysis[filter].

The search was conducted 26th of April 2021 and 222 references were evaluated.
