# Supplementary material for: Estimating impact of food choices on life expectancy: A modeling study
Source: PLoS Med. 2022 Feb 8;19(2):e1003889. doi: 10.1371/journal.pmed.1003889 (PMC8824353; doi:10.1371/journal.pmed.1003889)
Supplement: S3 Text — (PDF) [file pmed.1003889.s003.pdf]

S3 Text: Estimated intake of various food groups in the United States and Norway.

United States:
Vegetables: 249 g
Fruits: 184 g
Nuts: 11 g
Legumes: 9 g
Fish: 17 g
Egg: 44 g
Milk/dairy: 434 g
Whole grains (fresh weight): 30 g
Refined grains: 159 g
Meat, red: 95 g
Meat, processed: 53 g
Meat, white 75 g
Sugar sweetened beverages: 621 g
Added oils: 21 g

Norway:
Vegetables: 255 g (typical energy 2 kJ/g [mixed vegetables and potatoes])
Fruits: 244 g (typical energy 1.5 kJ/g [orange, raw])
Nuts: 11 g (typical energy 26 kJ/g [pea nuts, roasted and salted])
Legumes: 6 g (typical energy 5 kJ/g [chick peas, cooked])
Fish: 82 g (typical energy 6 kJ/g [mackerel, simmered])
Egg: 36 g (typical energy 6 kJ/g [boiled egg)
Milk/dairy: 225 g (typical energy 1.7 kJ/g [milk, 1% fat)
Whole grains (fresh weight): 61 g (typical energy 4 kJ/g [oatmeal porridge with water])
Refined grains: 172 g (typical energy 11 kJ/g [loaf bread])
Meat, red: 71 g (typical energy 7 kJ/g [cutlet])
Meat, processed: 58 g (typical energy 19 kJ/g [bacon])
Meat, white 58 g (typical energy 10 kJ/g [chicken leg])
Sugar sweetened beverages: 425 g (typical energy 2 kJ/g [lemonade])
Added oils: 30 g (typical energy 37 kJ/g [rapeseed oil])

Sources:
https://naldc.nal.usda.gov/download/19453/PDF
https://health.gov/dietaryguidelines/2015/guidelines/chapter-2/a-closer-look-at-current-intakes-and-recommended-shifts/
https://www.ers.usda.gov/data-products/food-consumption-and-nutrient-intakes/food-consumption-and-nutrient-intakes/#Food%20Consumption%20Estimates
https://www.ncbi.nlm.nih.gov/pubmed/31234969
https://www.ncbi.nlm.nih.gov/pmc/articles/PMC3045642/pdf/nihms-253312.pdf
https://nutrition.bmj.com/content/early/2019/08/27/bmjnph-2019-000034
https://www.statista.com/statistics/722080/bean-consumption-us-by-type/
https://www.ncbi.nlm.nih.gov/pmc/articles/PMC3662243/
Helsedirektoratet, Utviklingen i norsk kosthold 2018: Matforsyningsstatistikk og forbruksundersøkelser (IS-2804), in IS-2804. 2019
https://www.animalia.no/no/samfunn/sa-mye-kjott-spiser-vi/
http://www.matvaretabellen.no/
