# Supplementary material for: Estimating impact of food choices on life expectancy: A modeling study
Source: PLoS Med. 2022 Feb 8;19(2):e1003889. doi: 10.1371/journal.pmed.1003889 (PMC8824353; doi:10.1371/journal.pmed.1003889)
Supplement: S1 Table — (PDF) [file pmed.1003889.s004.pdf]

Supplementary file for "Estimating impact of food choices on life expectancy: A modeling study"

S1 Table: Hazard ratios (with uncertainty intervals) for various food groups with uncertainty limits (orange/red labels). Reference intake and energy levels per food groups are also indicated.

	Intake in grams/day																				Standard diet		Optimized		Norway patterns		US patterns	
**Grams**	0	25	50	100	150	200	250	300	400	500	600	700	800	Uncertainty threshold	No estimate threshold	Publication	kJ/g	Examples of food	kJ/100 g	‰ / 100 kJ	Sliders start	Energy (kJ)	Optimized	Energy (kJ)	Norway, mean intake	Energy (kJ)	US, mean intake	Energy (kJ)
Whole grains	1	0,935	0,87	0,8	0,76	0,72	0,7	0,7	0,72	0,72	0,8	0,8	0,87	300		Aune. Whole grain consumpti	4	Oat meal,	400	0,50	50	200	250	1000	61	244	30	122
- Mid to u +/-1			0,01	0,02	0,03	0,04	0,05	0,06	0,08	0,1	0,12	0,14	0,16															
CIlower			0,86	0,78	0,73	0,68	0,65	0,64	0,64	0,62	0,68	0,66	0,71															
CIupper			0,88	0,82	0,79	0,76	0,75	0,76	0,8	0,82	0,92	0,94	1,03															
**Veg**	1	0,965	0,93	0,87	0,835	0,8	0,785	0,77	0,76	0,76	0,75	0,75	0,75	700		Aune. Fruit and vegetable inta	2	Salad inclu	200	0,50	250	500	450	900	255	510	249	497
- Mid to upper/lower			0,015	0,025	0,04	0,05	0,05	0,05	0,05	0,05	0,06	0,07	0,08															
**Fruit**	1	0,97	0,94	0,89	0,865	0,84	0,83	0,82	0,81	0,81	0,81	0,82	0,84	700		Aune suppl. 164 (F&veg)	1,5	Oranges, a	150	0,53	200	300	400	600	244	366	184	276
- Mid to upper/lower	0	0	0,005	0,01	0,015	0,02	0,025	0,03	0,035	0,04	0,05	0,06	0,07															
Nuts	1	0,84	0,86	0,9	0,95									50	200	Aune. Nut consumption and r	26	Pea nuts	2600	0,25	0	0	25	650	11	286	11	284
- Mid to upper/lower		0,02	0,04	0,08	0,12																							
Legumes	1			0,9		0,8		0,75	0,72	0,71	0,72			250	625	Schwingshackl. Food groups a	5	Boiled chi	500	0,20	0	0	200	1000	6	30	9	43
- Mid to upper/lower		0,015	0,03	0,05	0,075	0,1	0,125	0,15	0,2	0,25	0,3																	
Fish	1		0,95	0,93		0,9	0,88	0,86	0,86	0,88	0,9			300	625	Schwingshackl. Food groups a	6	Mackerel,	600	0,12	50	300	200	1200	82	492	17	99
- Mid to upper/lower		0,02	0,025	0,03	0,04	0,06	0,08	0,12	0,16	0,2	0,25																	
Egg	1	0,98	1,05	1,3		1,5								100	225	Schwingshackl. Food groups a	6	Egg, boilec	600	0,13	50	300	25	150	36	216	44	264
- Mid to upper/lower		0,06	0,06	0,07	0,1	0,15																						
Milk	1			0,985		0,97		0,98	0,99	1,015	1,04	1,065	1,09			Schwingshackl. Food groups a	1,7	Milk, 1% fa	170	0,09	300	510	200	340	225	383	434	737
- Mid to upper/lower		0,01	0,015	0,015	0,0175	0,02	0,02	0,02	0,02	0,025	0,03	0,035	0,04															
Refined grains	1		0,94	0,98	1,03	1,09	1,16	1,24	1,4	1,57				200	525	Schwingshackl. Food groups a	11	Bread with	1100	0,11	150	1650	50	550	172	1892	159	1753
- Mid to upper/lower		0,03	0,06	0,07	0,1	0,13	0,16	0,19	0,25	0,31																		
Meat, red	1	1,05	1,09	1,18	1,3	1,42	1,54	1,66	1,9	2,14	2,38			250	625	Schwingshackl. Food groups a	7	Cutlets, ch	700	-0,30	100	700	0	0	93	651	95	665
- Mid to upper/lower	0	0,005	0,01	0,02	0,035	0,05	0,07	0,12	0,17	0,22	0,3																	
Meat, processed	1	1,1	1,18	1,3	1,43	1,57	1,71	1,85	2,13	2,41	2,69			250	625	Schwingshackl. Food groups a	19	Bacon	1900	-0,15	50	950	0	0	73	1387	53	1003
- Mid to upper/lower	0	0,01	0,02	0,05	0,1	0,15	0,2	0,25	0,32	0,4	0,5																	
Meat, white	1	1	1	1	1	1	1	1	1					0	625	Abete. Association between t	10	Chicken le	1000	0,00	75	750	50	500	50	500	75	750
- Mid to upper/lower	0	0,05	0,1	0,2	0,25	0,3	0,35	0,4	0,45		0,2																	
Sugar sweetened bev	1	1,0075	1,015	1,03	1,045	1,06	1,07	1,08	1,1	1,12	1,14	1,16	1,18	400		Schwingshackl. Food groups a	2	Lemonade	200	-0,15	500	1000	0	0	425	850	621	1242
- Mid to upper/lower	0	0,0125	0,025	0,05	0,055	0,06	0,065	0,07	0,08	0,09	0,1	0,11	0,14															
Added oils	**1**	**1**	**1**	**1**										0	**225**	Abdelhamid, A.S., et al., Polyu	37	Rapeseed	3700	0,14	25	925	25	925	30	1110	21	777
- Mid to upper/lower	0	0,05	0,1	0,2																								
				**Orange label:** Uncertain estimates (moderate)					**Red label**: No estimate (very high uncertainty)							**Total estimates**					**1 800**	**8 085**		**7 815**	**1 763**	**8 917**		**8 512**
