# Supplementary material for: Estimating impact of food choices on life expectancy: A modeling study
Source: PLoS Med. 2022 Feb 8;19(2):e1003889. doi: 10.1371/journal.pmed.1003889 (PMC8824353; doi:10.1371/journal.pmed.1003889)
Supplement: S2 Table — FA, feasibility approach diet; LE, life expectancy; OD, optimized diet; TW, typical Western diet. (PDF) [file pmed.1003889.s005.pdf]

S2 Table: Increase in life expectancy (LE) for each food group change for 20- and 60-year-old female and male adults from the United States, who changes from a typical Western diet (TW) to optimized (OD) or feasibility approach (FA). Estimates per food groups and total change in life expectancy is presented with 95% uncertainty intervals (UI).

Food group	Diet			Life years gained for adults from the United States (95% UI)							
				20 years of age when initiating changes				60 years of age when initiating changes			
				Female		Male		Female		Male	
	TW. g/day	FA. g/day	OD. g/day	TW->FA	TW->OD	TW->FA	TW->OD	TW->FA	TW->OD	TW->FA	TW->OD
Legumes	0	100	200	1.1 (0.6;1.6)	2.2 (1.2;3.5)	1.2 (0.6;1.8)	2.5 (1.3;4.0)	0.8 (0.4;1.1)	1.6 (0.9;2.5)	0.8 (0.4;1.2)	1.6 (0.9;2.6)
Whole grains	50	137.5	225	1.2 (0.9;1.6)	2.0 (1.4;2.7)	1.4 (1.0;1.8)	2.3 (1.6;3.0)	0.9 (0.6;1.2)	1.4 (1.0;1.9)	0.9 (0.7;1.2)	1.5 (1.1;2.0)
Nuts	0	12.5	25	0.8 (0.7;0.9)	1.7 (1.5;2.0)	1.0 (0.8;1.1)	2.0 (1.7;2.3)	0.6 (0.5;0.7)	1.2 (1.1;1.4)	0.6 (0.5;0.7)	1.3 (1.1;1.5)
Fish	50	125	200	0.3 (-0.2;0.8)	0.5 (-0.2;1.3)	0.3 (-0.3;0.9)	0.6 (-0.2;1.5)	0.2 (-0.1;0.6)	0.4 (-0.1;0.9)	0.2 (-0.1;0.6)	0.4 (-0.1;0.9)
Fruits	200	300	400	0.2 (-0.2;0.7)	0.4 (-0.1;0.9)	0.3 (-0.3;0.8)	0.4 (-0.2;1.0)	0.2 (-0.1;0.5)	0.3 (-0.1;0.6)	0.2 (-0.2;0.6)	0.3 (-0.1;0.6)
Vegetables	250	325	400	0.2 (-0.9;1.3)	0.3 (-0.6;1.4)	0.3 (-1.0;1.3)	0.4 (-0.9;1.5)	0.2 (-0.7;0.9)	0.2 (-0.5;1.0)	0.2 (-0.5;1.0)	0.2 (-0.4;1.1)
White meat	75	62.5	50	0.0 (-2.3;1.8)	0.0 (-2.0;1.7)	0.0 (-2.4;2.4)	0.0 (-2.7;2.1)	0.0 (-1.7;1.4)	0.0 (-1.5;1.5)	0.0 (-1.6;1.4)	0.0 (-1.7;1.5)
Milk/dairy	300	250	200	0.1 (-0.3;0.4)	0.1 (-0.2;0.4)	0.1 (-0.3;0.5)	0.1 (-0.2;0.5)	0.0 (-0.2;0.3)	0.1 (-0.1;0.3)	0.0 (-0.2;0.3)	0.1 (-0.1;0.3)
Eggs	50	37.5	25	0.3 (-0.6;1.3)	0.7 (-0.2;1.7)	0.4 (-0.6;1.4)	0.8 (-0.2;1.9)	0.2 (-0.4;0.9)	0.5 (-0.2;1.1)	0.3 (-0.3;0.9)	0.5 (-0.2;1.2)
Refined grains	150	100	50	0.5 (-0.7;1.8)	0.9 (-0.3;2.2)	0.6 (-1.0;2.2)	1.1 (-0.5;2.5)	0.4 (-0.6;1.4)	0.7 (-0.3;1.5)	0.4 (-0.7;1.2)	0.7 (-0.3;1.5)
Sugar-sweetened beverages	500	250	0	0.5 (-0.7;1.6)	1.1 (0.3;1.8)	0.5 (-0.7;1.8)	1.3 (0.3;2.1)	0.3 (-0.5;1.0)	0.8 (0.2;1.3)	0.3 (-0.4;1.1)	0.8 (0.2;1.4)
Red meat	100	50	0	0.8 (0.6;1.0)	1.6 (1.5;1.8)	0.9 (0.7;1.1)	1.9 (1.7;2.1)	0.6 (0.4;0.7)	1.2 (1.1;1.3)	0.6 (0.4;0.7)	1.2 (1.1;1.3)
Processed meat	50	25	0	0.7 (0.5;0.9)	1.6 (1.5;1.8)	0.8 (0.6;1.1)	1.9 (1.7;2.1)	0.5 (0.4;0.7)	1.2 (1.1;1.3)	0.5 (0.4;0.7)	1.2 (1.1;1.3)
**Total**				**6.2 (3.5;8.1)**	**10.7 (8.4;12.3)**	**7.3 (4.7;9.5)**	**13.0 (9.4;14.3)**	**4.5 (2.7;6.1)**	**8.0 (6.2;9.3)**	**4.8 (3;6.4)**	**8.8 (6.8;10.0)**

* Uncertainty intervals for some food groups have rounding differences in the last digit compared to corresponding figures due to figures are automatically adjusted to be symmetrical through the admetan package in Stata..
