# Supplementary material for: Estimating impact of food choices on life expectancy: A modeling study
Source: PLoS Med. 2022 Feb 8;19(2):e1003889. doi: 10.1371/journal.pmed.1003889 (PMC8824353; doi:10.1371/journal.pmed.1003889)
Supplement: S3 Table — LE, life expectancy. (PDF) [file pmed.1003889.s006.pdf]

S3 Table: Absolute and relative change in life expectancy with delay to full effects of 10 (default), 5, 30 and 50 years for 20-, 40-, 60- and 80-year-old females and males from the United States.

			**Absolute change (years)**				**Relative change (proportion)**			
Age	Sex	Estimate	10-year	5-year	30-year	50-year	10-year	5-year	30-year	50-year
20	Female	10.7	0	0.1	-0.4	-1.3	0	0.01	-0.04	-0.12
20	Male	13.0	0	0.2	-0.9	-2.2	0	0.02	-0.07	-0.17
40	Female	10.0	0	0.2	-1.2	-3.4	0	0.02	-0.12	-0.34
40	Male	11.7	0	0.3	-1.8	-4.7	0	0.03	-0.15	-0.40
60	Female	8.0	0	0.5	-2.8	-5.3	0	0.06	-0.35	-0.66
60	Male	8.8	0	0.8	-3.6	-6.1	0	0.09	-0.41	-0.69
80	Female	3.4	0	1.2	-2.4	-2.8	0	0.35	-0.71	-0.82
80	Male	3.4	0	1.3	-2.4	-2.9	0	0.38	-0.71	-0.85
