# Supplementary material for: Estimating impact of food choices on life expectancy: A modeling study
Source: PLoS Med. 2022 Feb 8;19(2):e1003889. doi: 10.1371/journal.pmed.1003889 (PMC8824353; doi:10.1371/journal.pmed.1003889)

Supplementary file for "Estimating impact of food choices on life expectancy: A modeling study"

S1 Fig: Example of calculator input and output for the Food4HealthyLife calculator exemplified for 20-year-old female from Europe based on change from a typical Western (left food panel) to a feasibility-approach diet (right food panel). Health gain per food group is presented in a forest plot above the food input and total life expectancy change below (see http://food4healthylife.org/).

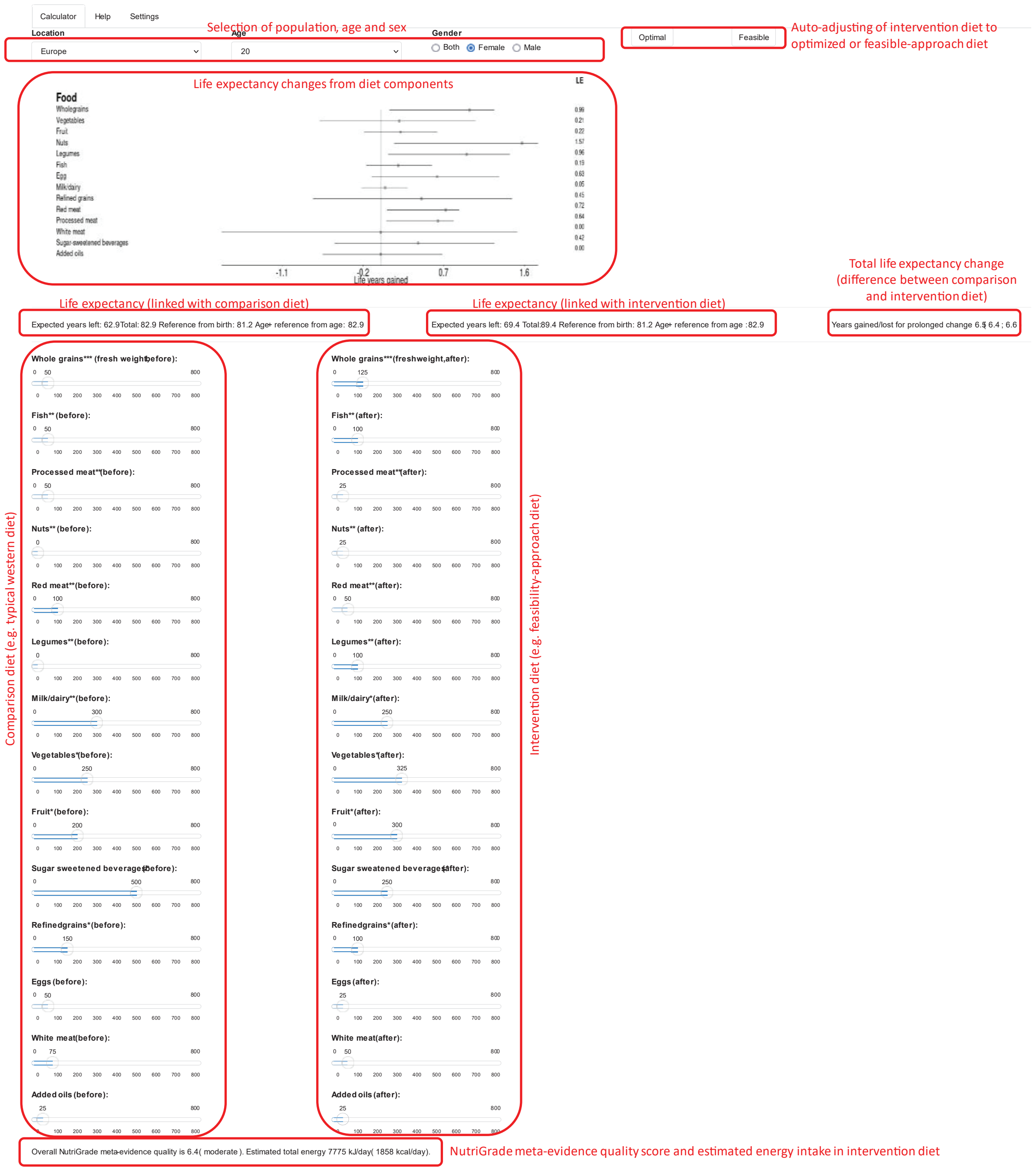

Supplement: S1 Fig — (PDF) [file pmed.1003889.s007.pdf]
