# Supplementary figures and images for: Estimating impact of food choices on life expectancy: A modeling study
Source: PLoS Med. 2022 Feb 8;19(2):e1003889. doi: 10.1371/journal.pmed.1003889 (PMC8824353; doi:10.1371/journal.pmed.1003889)

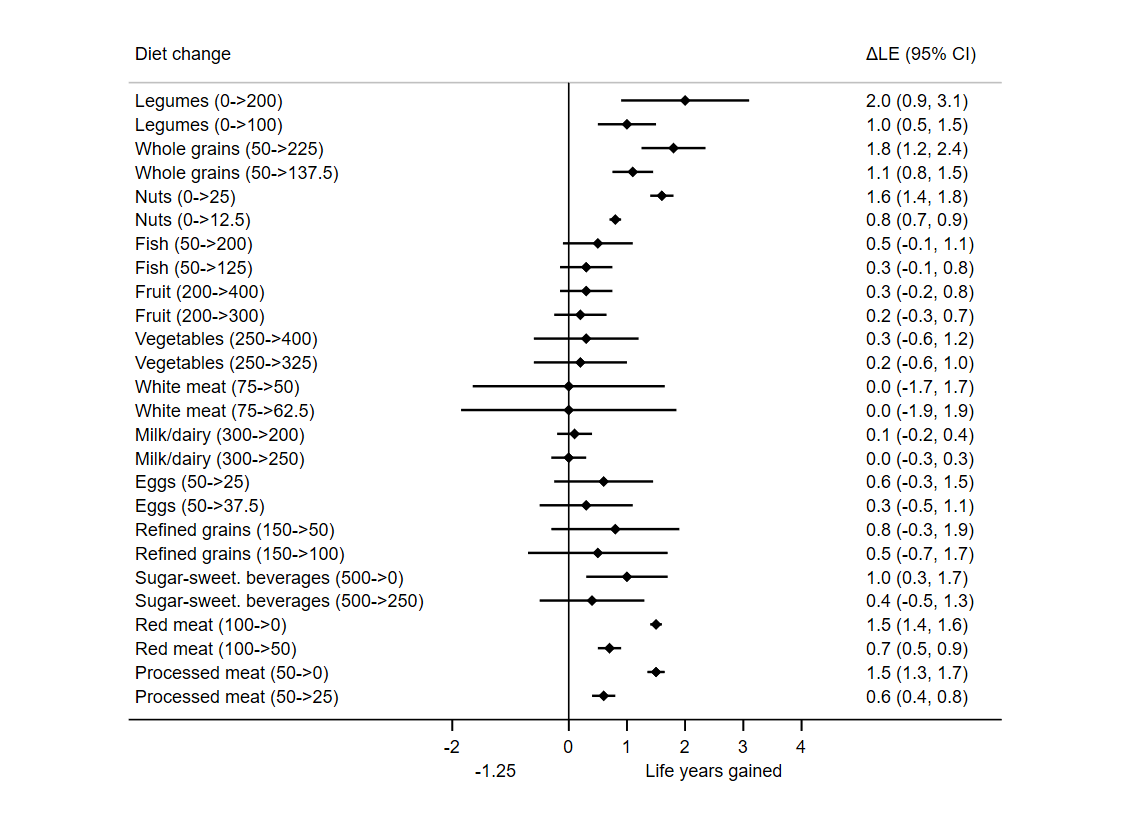

Supplement: S2 Fig — (PNG) [file pmed.1003889.s008.png]

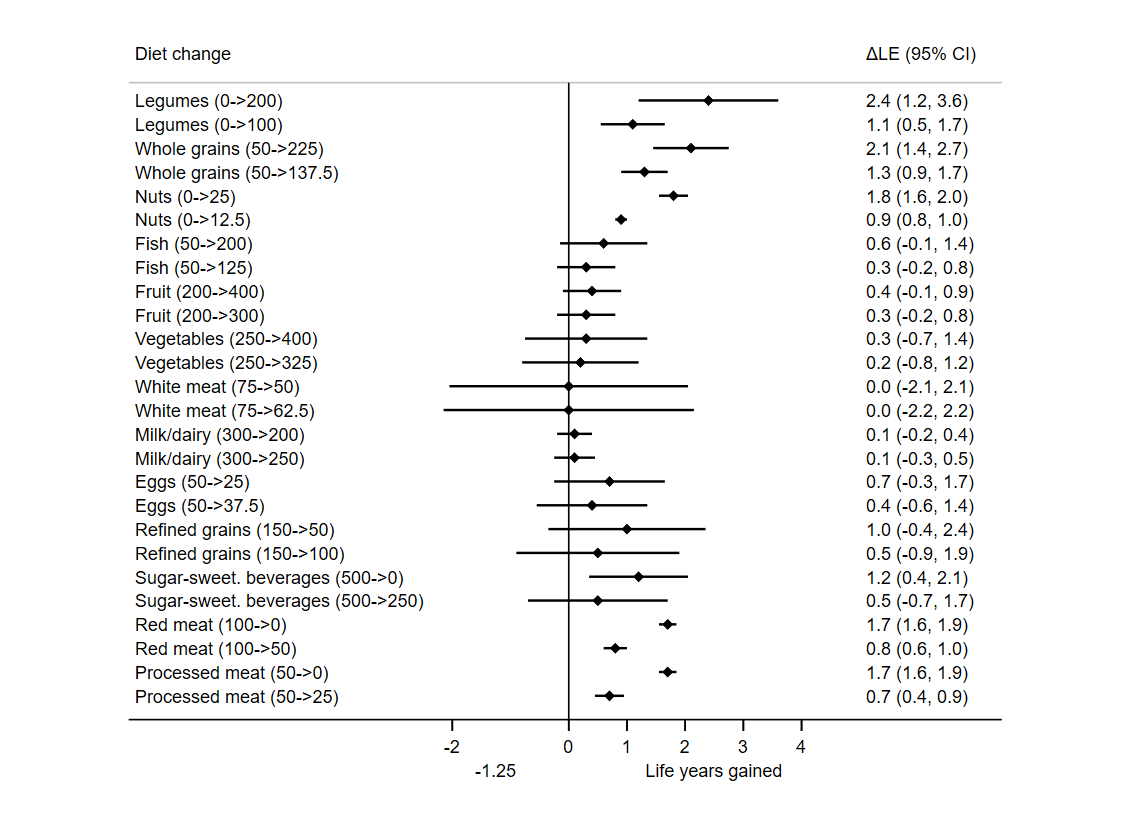

Supplement: S3 Fig — Estimates per food group and change in LE are presented with 95% UIs. LE, life expectancy; 95% UI, 95% uncertainty interval. (PNG) [file pmed.1003889.s009.png]

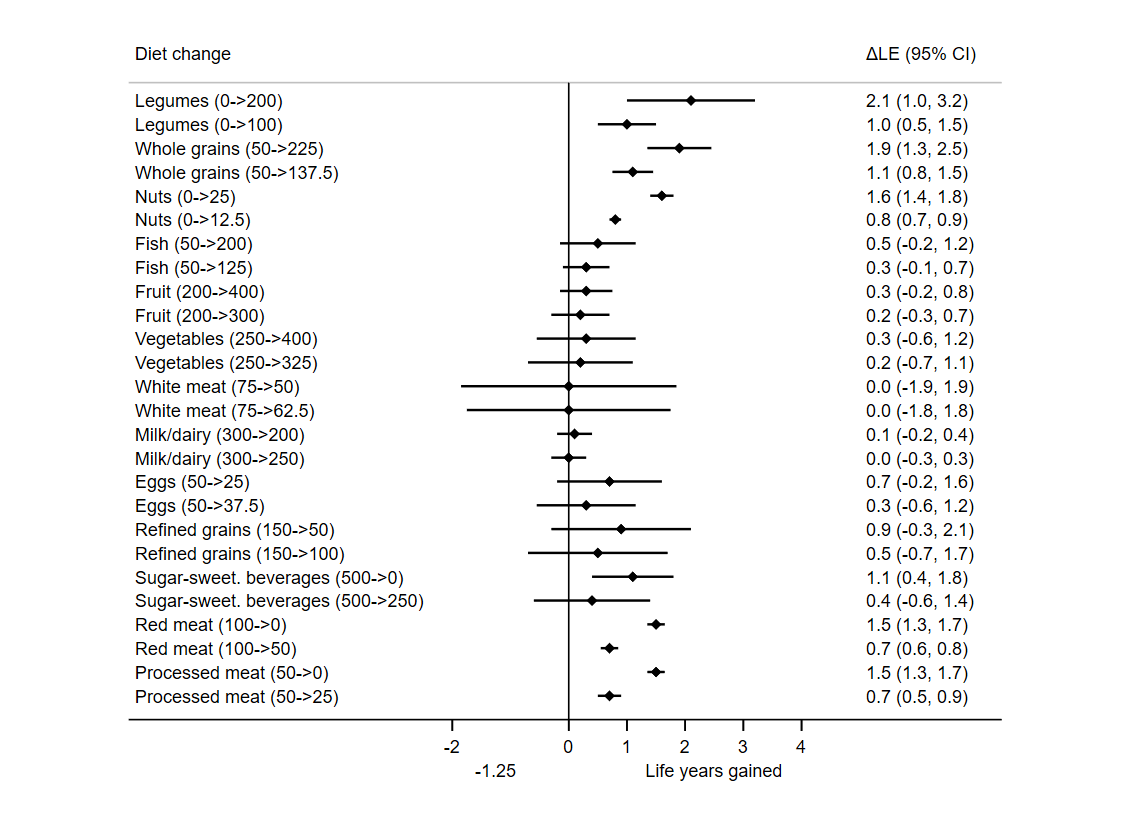

Supplement: S4 Fig — Estimates per food group and change in LE are presented with 95% UIs. LE, life expectancy; 95% UI, 95% uncertainty interval. (PNG) [file pmed.1003889.s010.png]

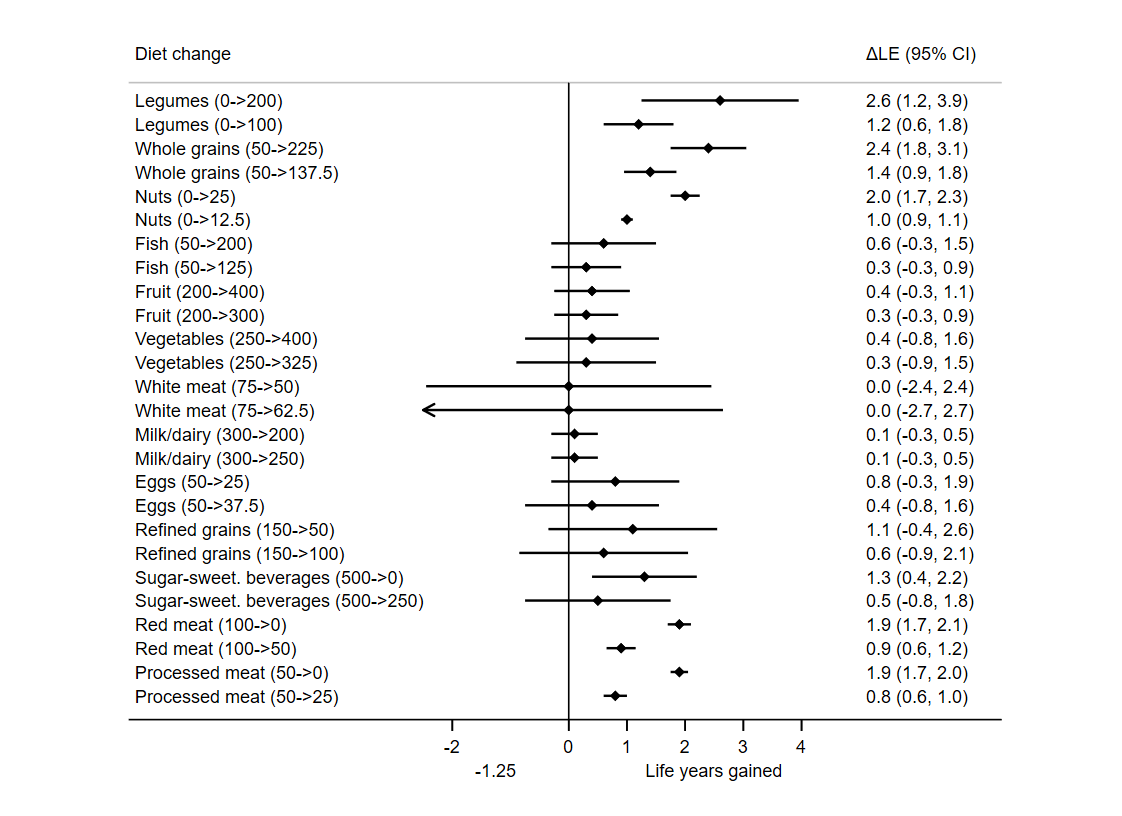

Supplement: S5 Fig — Estimates per food group and change in LE are presented with 95% UIs. LE, life expectancy; 95% UI, 95% uncertainty interval. (PNG) [file pmed.1003889.s011.png]

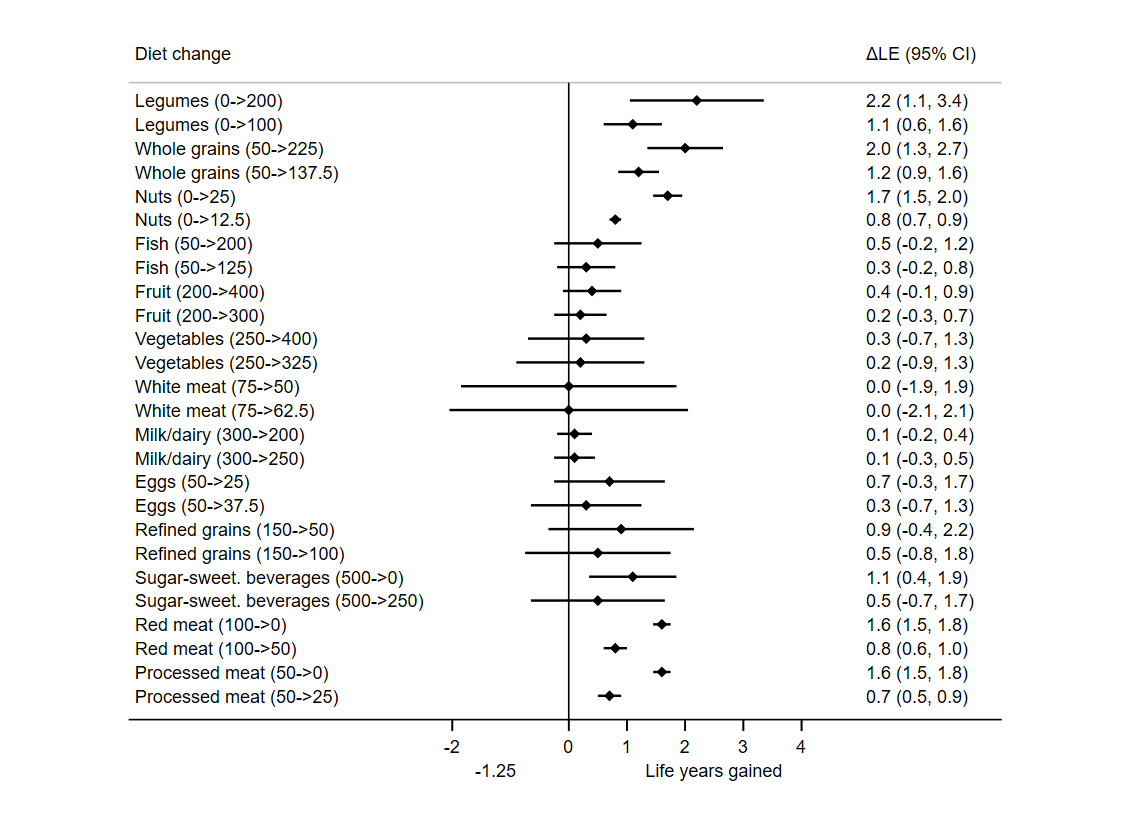

Supplement: S6 Fig — Estimates per food group and change in LE are presented with 95% UIs. LE, life expectancy; 95% UI, 95% uncertainty interval. (PNG) [file pmed.1003889.s012.png]

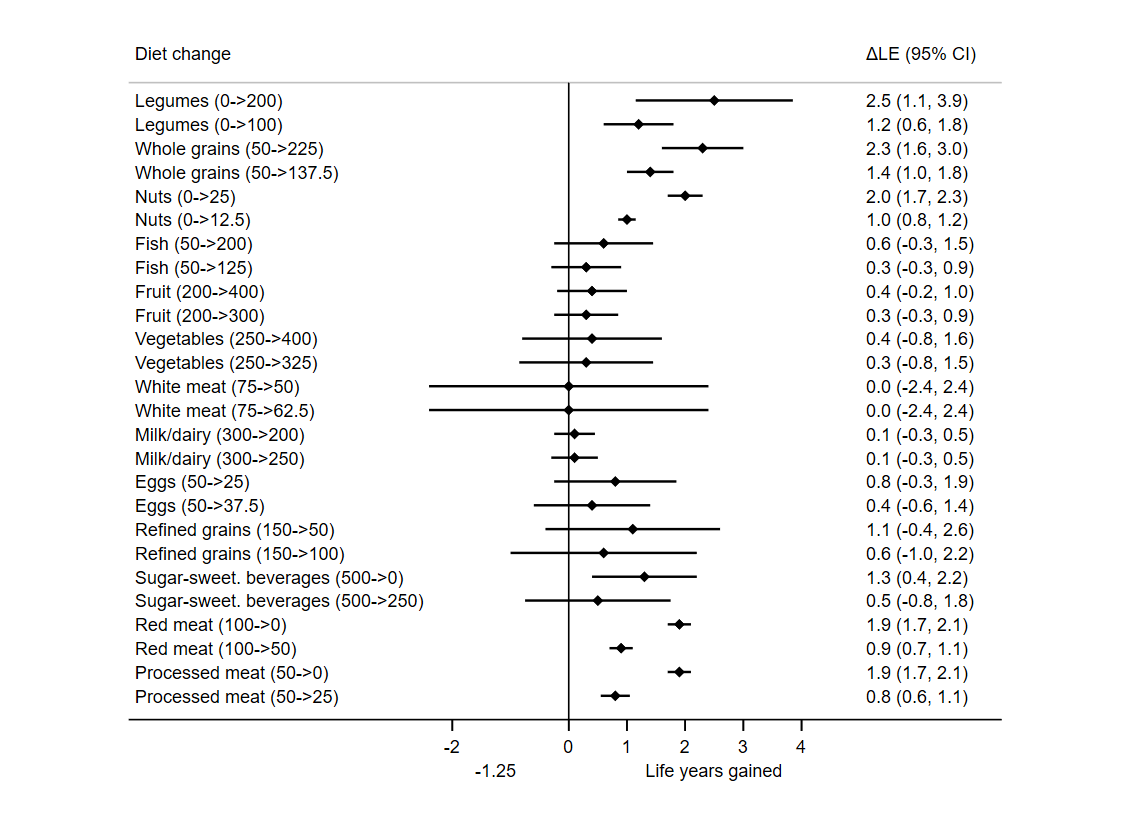

Supplement: S7 Fig — Estimates per food group and change in LE are presented with 95% UIs. LE, life expectancy; 95% UI, 95% uncertainty interval. (PNG) [file pmed.1003889.s013.png]

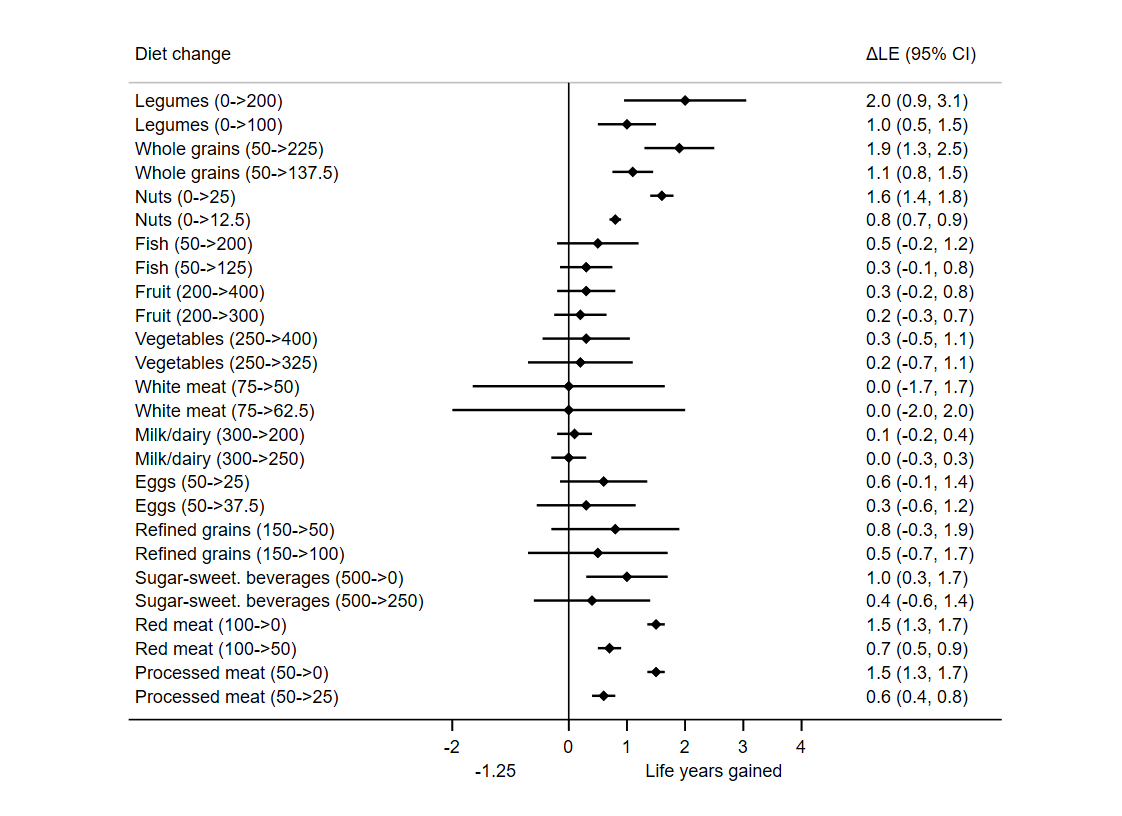

Supplement: S8 Fig — Estimates per food group and change in LE are presented with 95% UIs. LE, life expectancy; 95% UI, 95% uncertainty interval. (PNG) [file pmed.1003889.s014.png]

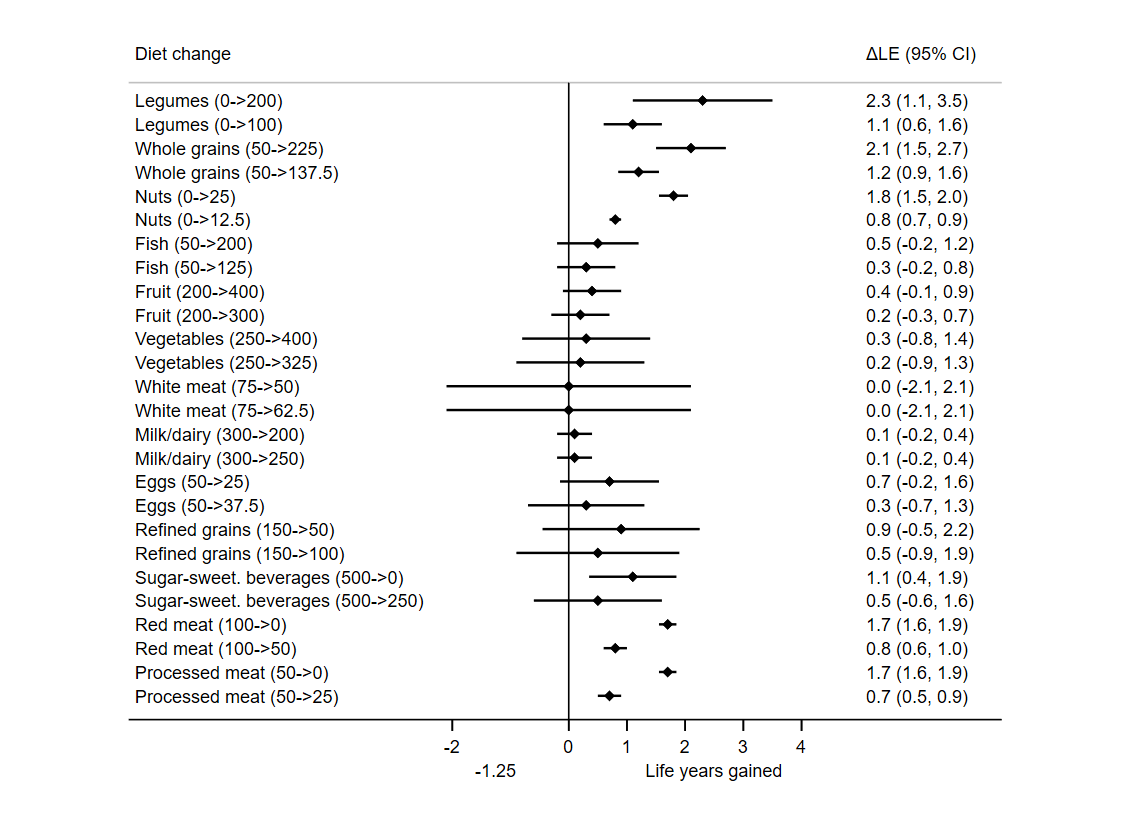

Supplement: S9 Fig — Estimates per food group and change in LE are presented with 95% UIs. LE, life expectancy; 95% UI, 95% uncertainty interval. (PNG) [file pmed.1003889.s015.png]

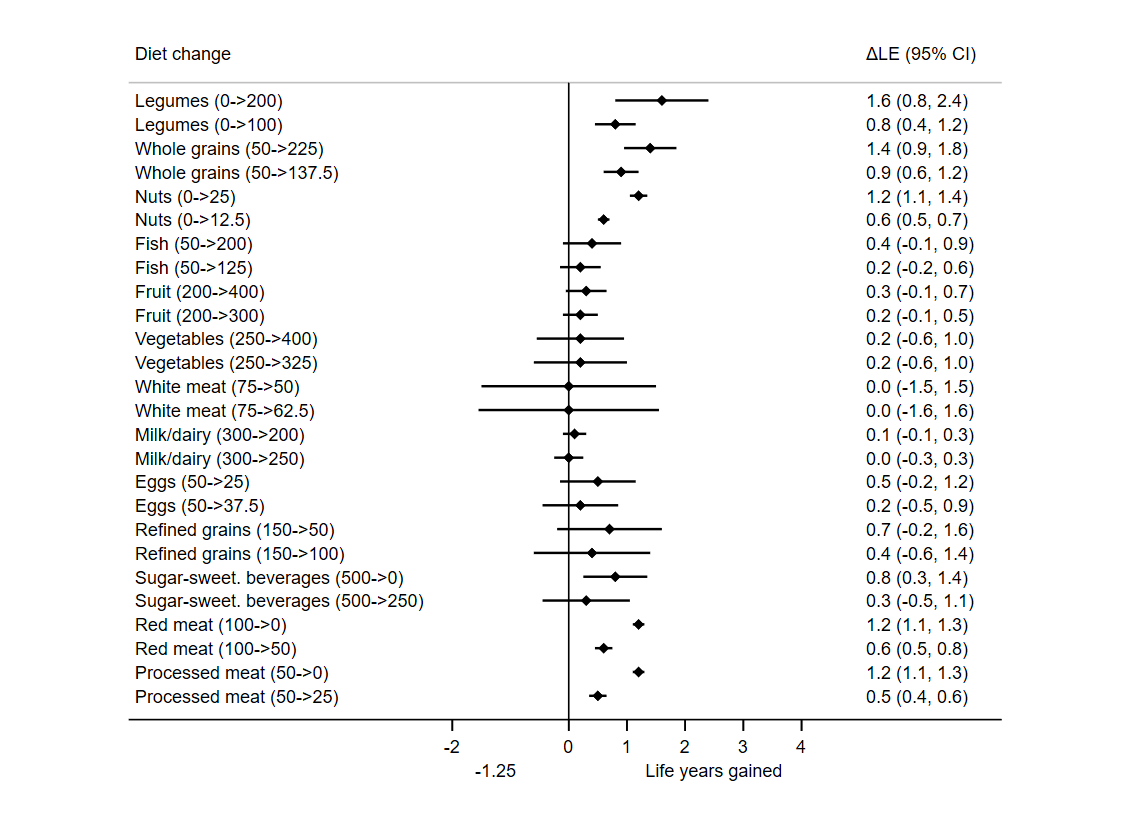

Supplement: S10 Fig — Estimates per food group and change in LE are presented with 95% UIs. LE, life expectancy; 95% UI, 95% uncertainty interval. (PNG) [file pmed.1003889.s016.png]

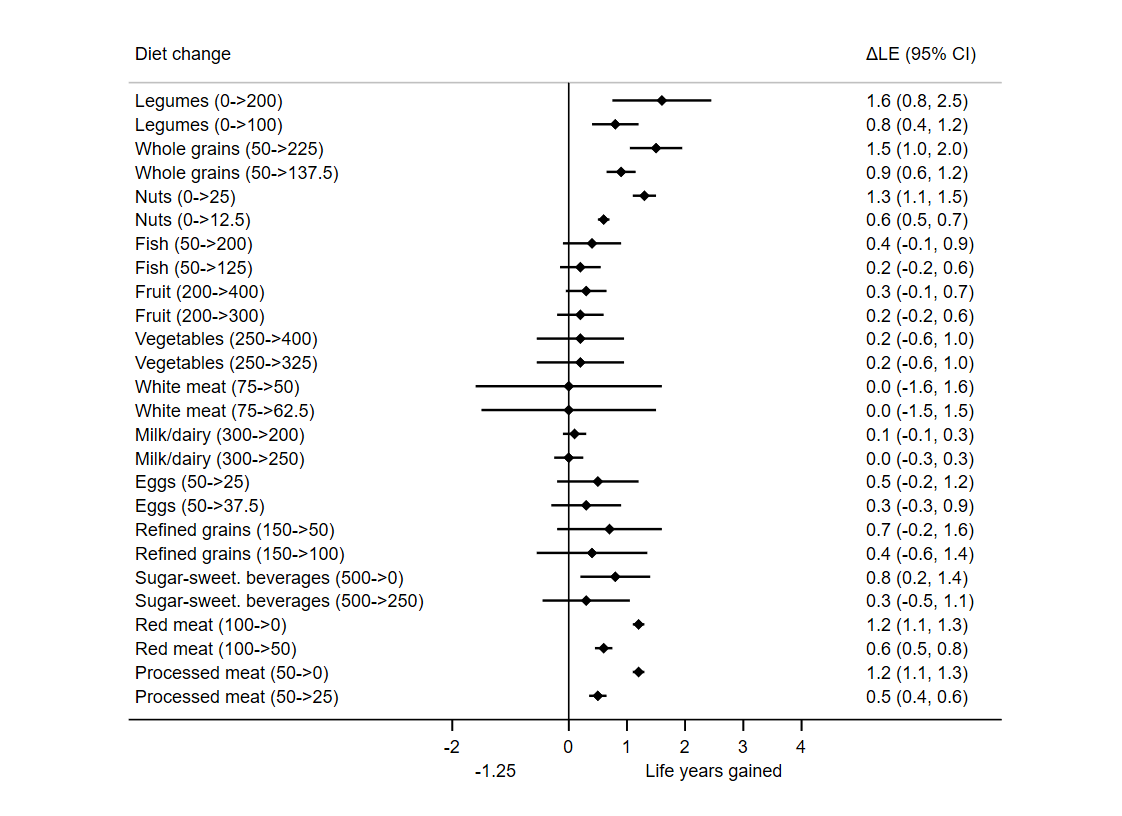

Supplement: S11 Fig — Estimates per food group and change in LE are presented with 95% UIs. LE, life expectancy; 95% UI, 95% uncertainty interval. (PNG) [file pmed.1003889.s017.png]

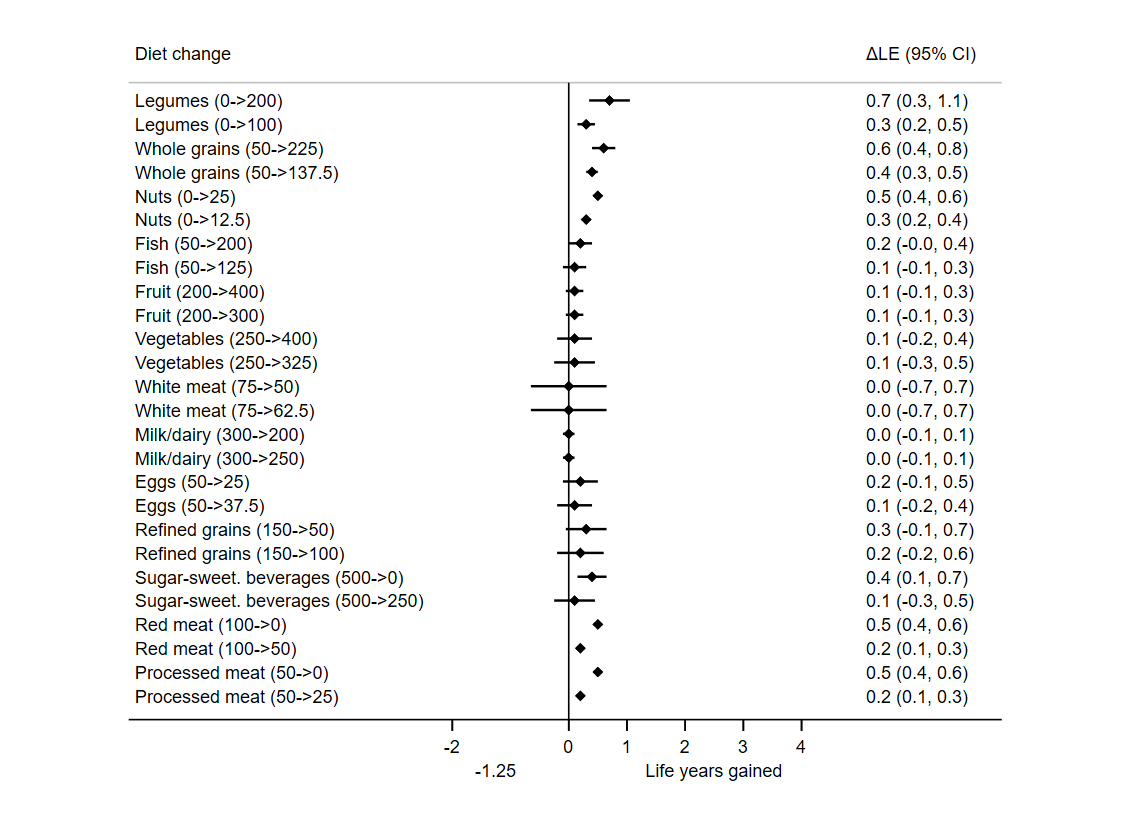

Supplement: S12 Fig — Estimates per food group and change in LE are presented with 95% UIs. LE, life expectancy; 95% UI, 95% uncertainty interval. (PNG) [file pmed.1003889.s018.png]

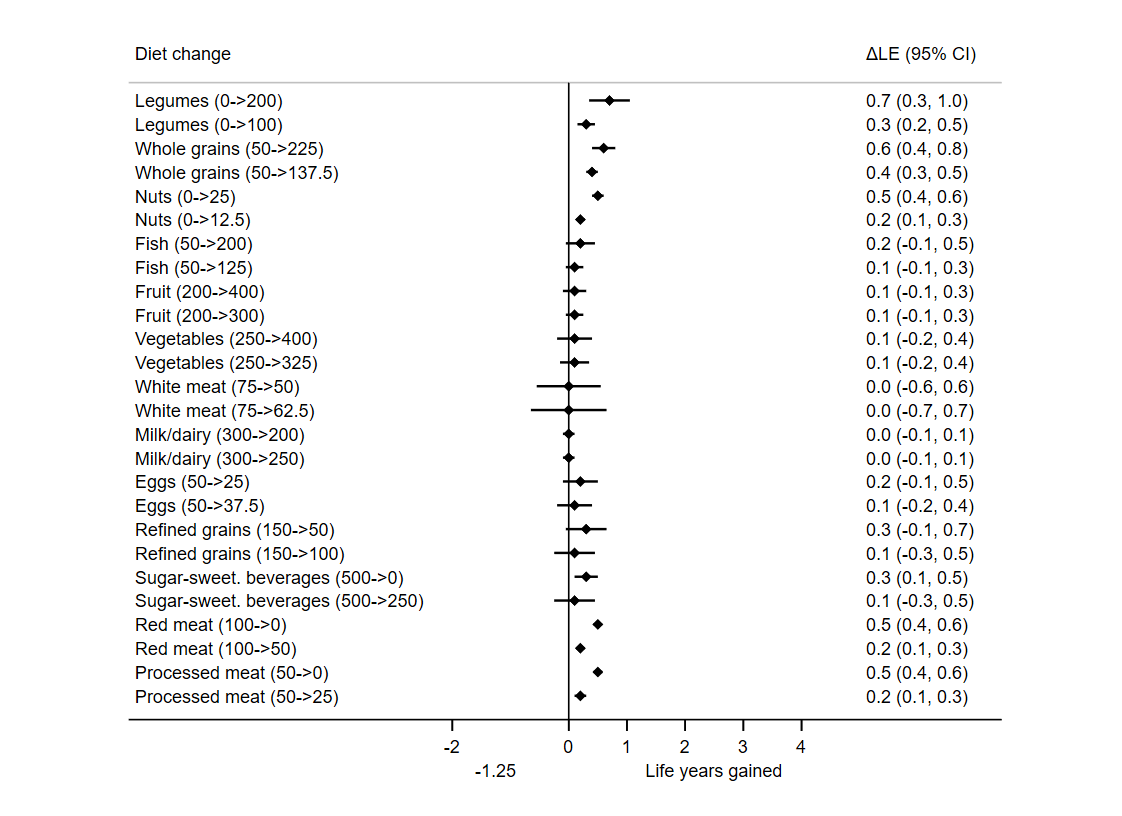

Supplement: S13 Fig — Estimates per food group and change in LE are presented with 95% UIs. LE, life expectancy; 95% UI, 95% uncertainty interval. (PNG) [file pmed.1003889.s019.png]

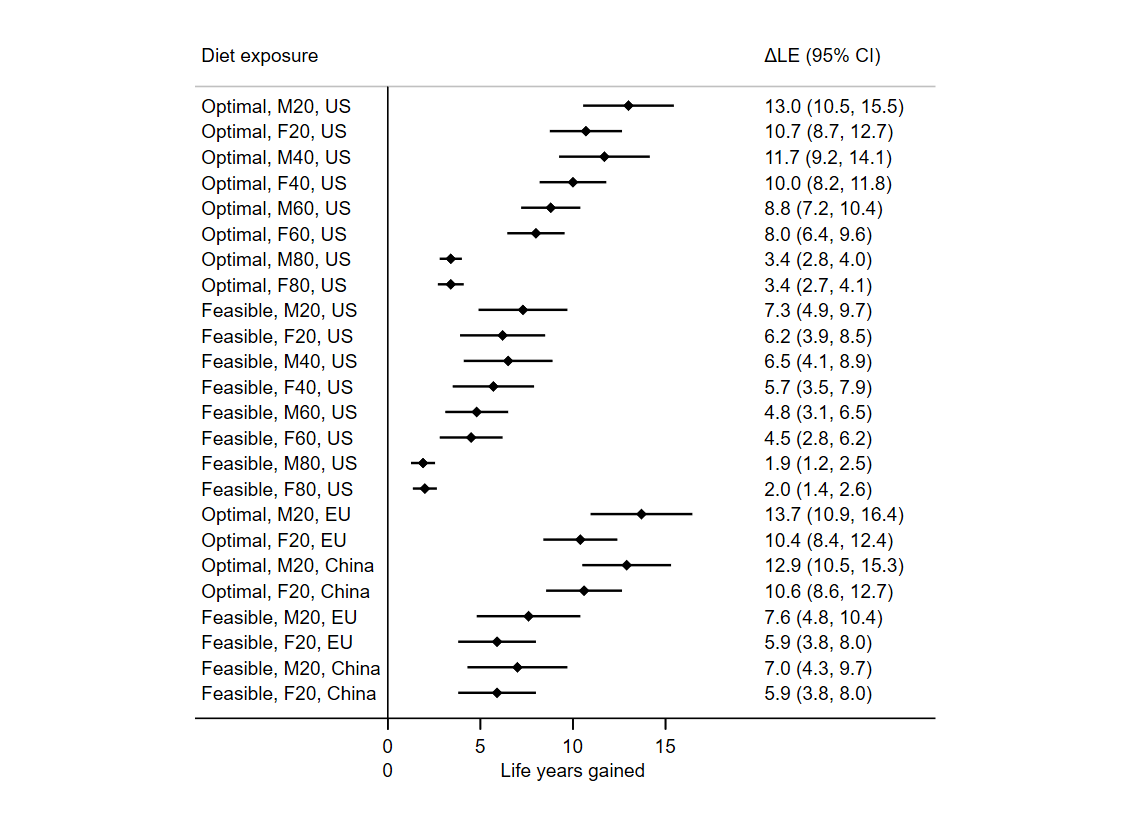

Supplement: S14 Fig — Estimates for change in LE is presented with 95% UIs. *For the optimal diet and feasibility approach diet, the following intakes were used: 225/137.5 g whole grains (fresh weight), 400/325 g vegetables, 400/300 g fruits, 25/12.5 g nuts, 200/100 g legumes, 200/125 g fish, 25/37.5 g eggs, 200/250 g milk/dairy, 50/100 g refined grains, 0/50 g red meat, 0/25 g processed meat, 50/62.5 g white meat, 0/250 g sugar-sweetened beverages, and 25/25 g added plant oils. **F20 indicates 20-year-old females, and M60 indicates 60-year-old males. Uncertainty intervals for some food groups have rounding differences compared to corresponding S2 Table due to symmetrical adjustment in the admetan package in Stata. EU, Europe; LE, life expectancy; US, United States; 95% UI, 95% uncertainty interval. (PNG) [file pmed.1003889.s020.png]

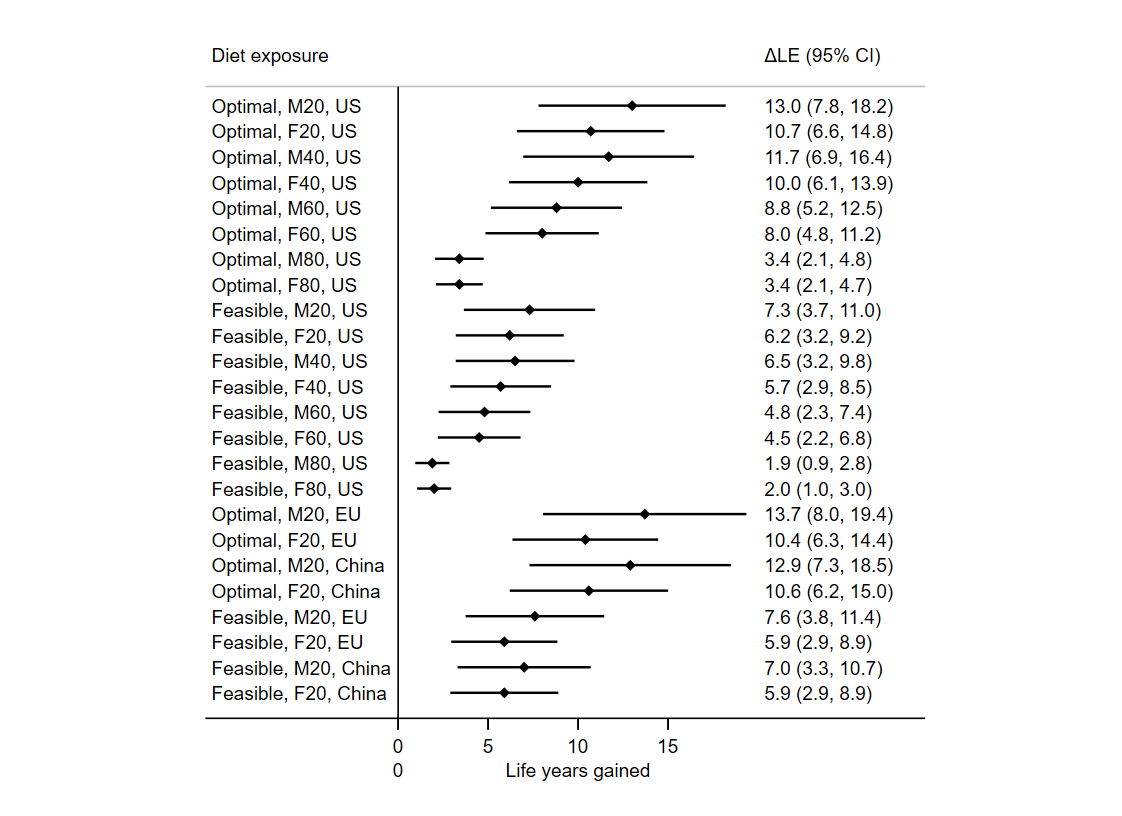

Supplement: S15 Fig — Estimates for change in LE is presented with sensitivity adjusted uncertainty intervals using lower interval as model adjustment of 0.5 and upper interval as model adjustment of 1.5. *For the optimal diet and feasibility approach diet, the following intakes were used: 225/137.5 g whole grains (fresh weight), 400/325 g vegetables, 400/300 g fruits, 25/12.5 g nuts, 200/100 g legumes, 200/125 g fish, 25/37.5 g eggs, 200/250 g milk/dairy, 50/100 g refined grains, 0/50 g red meat, 0/25 g processed meat, 50/62.5 g white meat, 0/250 g sugar-sweetened beverages, and 25/25 g added plant oils. **F20 indicates 20-year-old females, and M60 indicates 60-year-old males. Uncertainty intervals for some food groups have rounding differences compared to corresponding S2 Table due to symmetrical adjustment in the admetan package in Stata. EU, Europe; LE, life expectancy; US, United States. (PNG) [file pmed.1003889.s021.png]

## Optimal

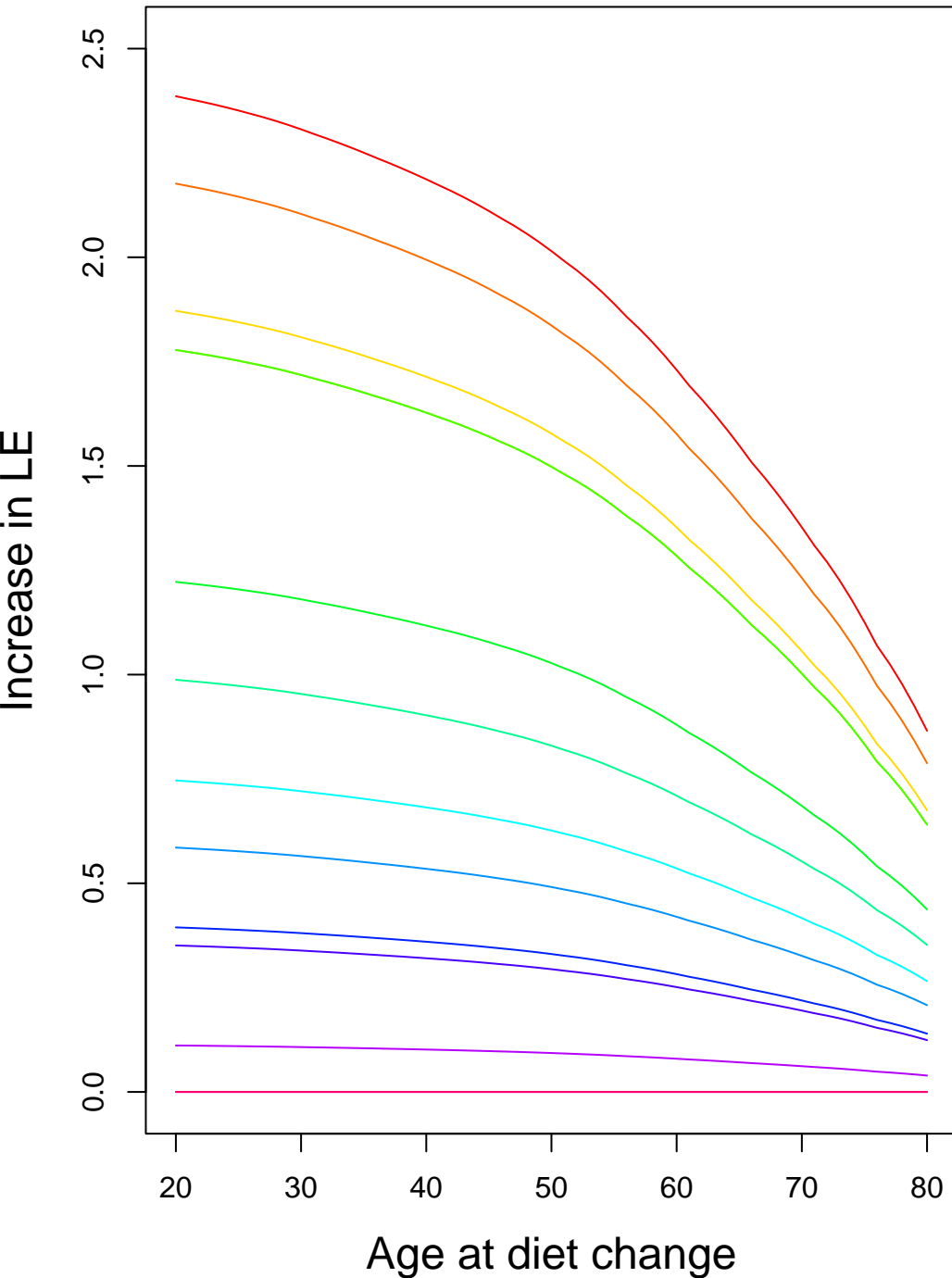

## Feasible

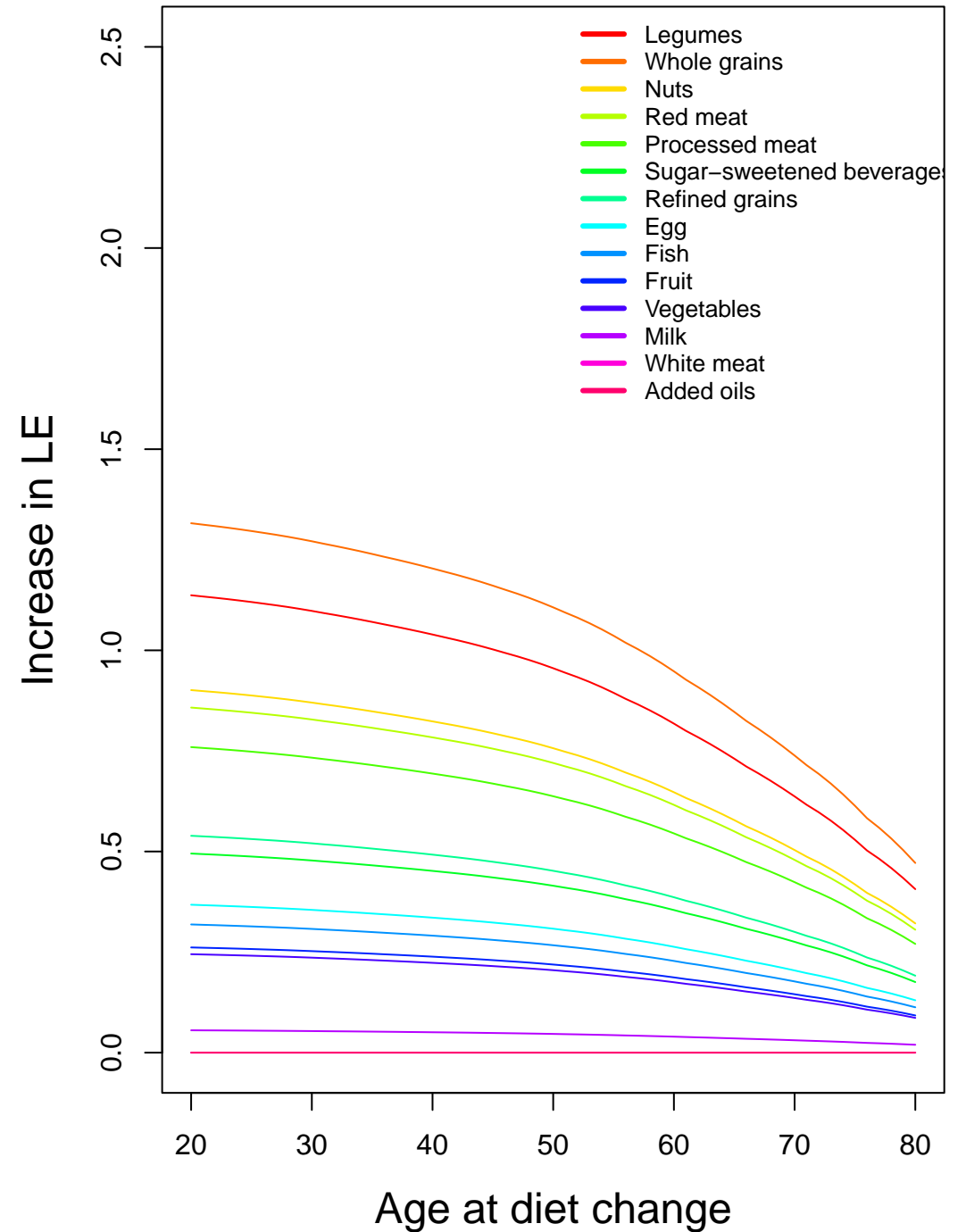

Supplement: S16 Fig — *For the optimal diet and feasibility approach diet, the following intakes were used: 225 g and 137.5 g whole grains (fresh weight), 400 g and 325 g vegetables, 400 g and/ 300 g fruits, 25 g and 12.5 g nuts, 200 g and/ 100 g legumes, 200 g and 100 g fish, 25 g and 37.5 g eggs, 200 g and 250 g milk/dairy, 50 g and 100 g refined grains, 0 g and 50 g red meat, 0 g and 25 g processed meat, 50 g and 62.5 g white meat, 0 g and 250 g sugar-sweetened beverages, and 25 g and 25 g added plant oils. LE, life expectancy. (PDF) [file pmed.1003889.s022.pdf]

## Optimal

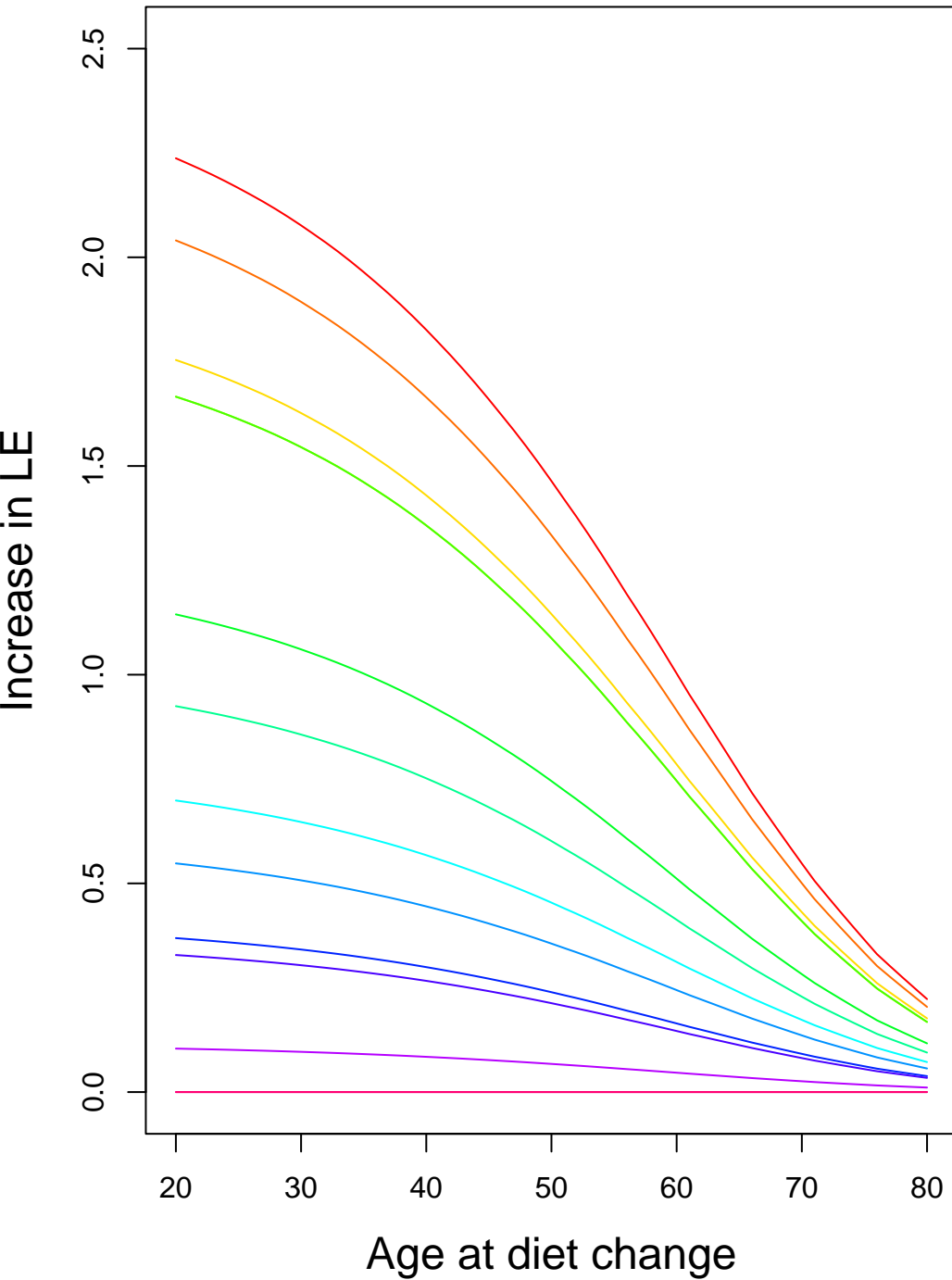

## Feasible

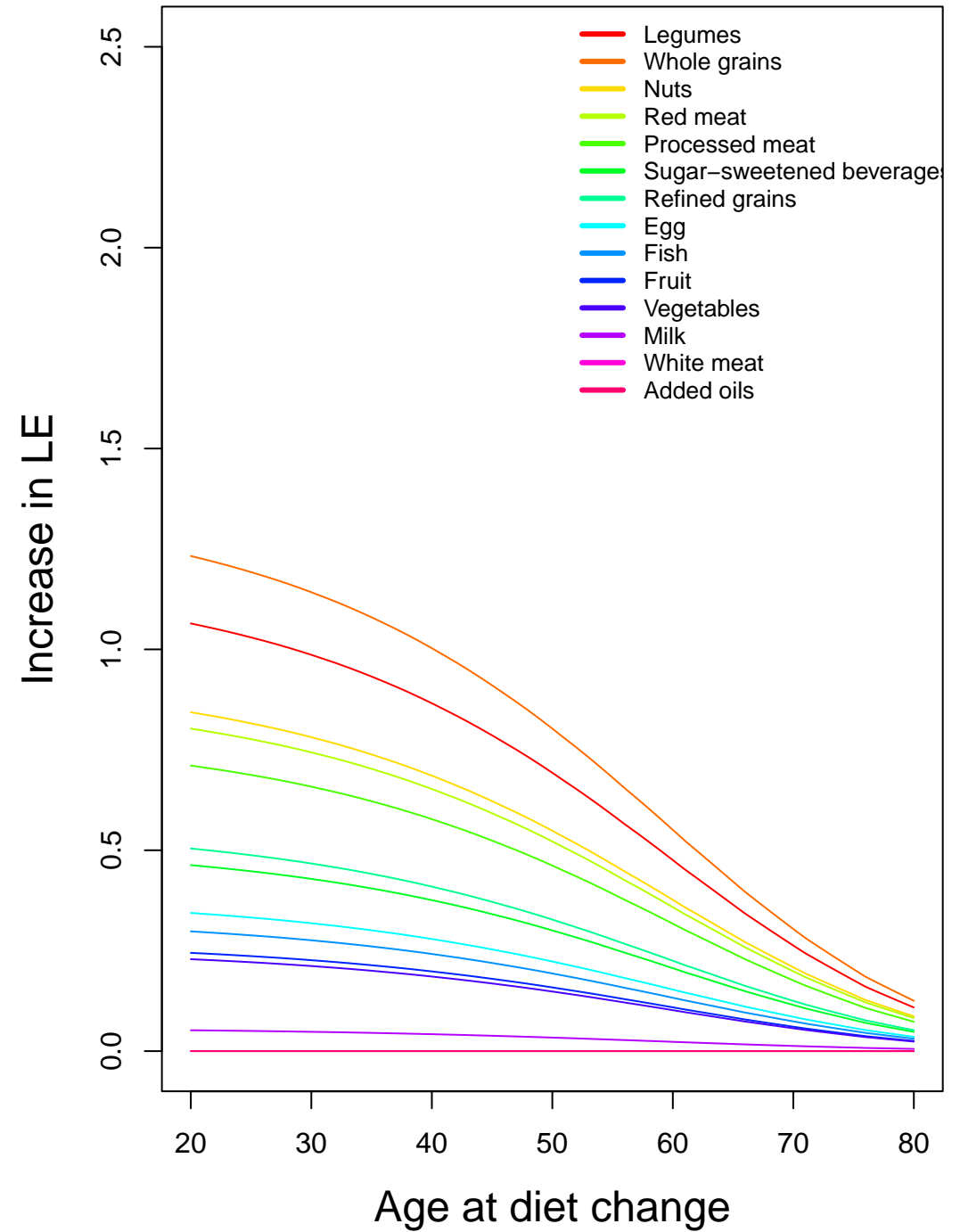

Supplement: S17 Fig — *For the optimal diet and feasibility approach diet, the following intakes were used: 225 g and 137.5 g whole grains (fresh weight), 400 g and 325 g vegetables, 400 g and/ 300 g fruits, 25 g and 12.5 g nuts, 200 g and/ 100 g legumes, 200 g and 100 g fish, 25 g and 37.5 g eggs, 200 g and 250 g milk/dairy, 50 g and 100 g refined grains, 0 g and 50 g red meat, 0 g and 25 g processed meat, 50 g and 62.5 g white meat, 0 g and 250 g sugar-sweetened beverages, and 25 g and 25 g added plant oils. LE, life expectancy. (PDF) [file pmed.1003889.s023.pdf]
