# Supplementary material for: Estimating impact of food choices on life expectancy: A modeling study
Source: PLoS Med. 2022 Feb 8;19(2):e1003889. doi: 10.1371/journal.pmed.1003889 (PMC8824353; doi:10.1371/journal.pmed.1003889)
Supplement: Attachment — Submitted filename: Response-reviewer-3-R1-F4HL-PLOS Medicine.pdf [file pmed.1003889.s025.pdf]

**** Reviewer #3:*

Alex McConnachie, Statistical Review

The paper by Fadnes et al looks at modelling the possible impact of sustained dietary changes from a western-style diet towards a more optimal diet, on life expectancy. This review considers the statistical elements of the paper.

On the one hand, this could be a very short review, since there are no statistical methods used in the paper, in the usual sense of hypothesis testing and statistical model fitting. Nevertheless, I read the paper with interest, and I have a few comments that I hope will improve the paper.

The descriptions of a typical western diet, the optimal diet, and the feasibility-approach diet were nice and clear. The idea of the FA diet lying half-way between the typical and OD was a good one, as an attempt to reflect what might be practically achievable by most people, though I thought some of the calculations looked wrong. For example, if the typical intake of nuts is taken as zero, and is 25g for the OD, should it be 12.5g (not 25g) for FA? For fish, should it be 125g (not 100g) for fish (midway between 50 and 200g)? The values for the FA diet do not seem to match the way it was described.

> **Response:** Thanks for these comments. We agree that it would be more sensible to choose 12.5g for nuts and 125g for fish as feasible-approach diet and have implemented these changes. We similarly also modified white meat and eggs. To allow for exact mid-point of these food items, we changed the increment on the food items from 25g to 12.5g.

The simulation approach looks OK, but was only repeated 200 times for each scenario. Is this enough? Whenever I use a sampling-based method, I tend to use thousands of replications - nowadays, computing power is not an issue with these things, so I think it is worth erring on the high side.

> **Response:** We see your point that more is generally better in terms of similation iterations. However, we observed a notable delay in the time to receive output when changing from 200 to e.g. 1000 iterations (with many seconds delay as there are relatively long command lines that needs to be repeated), without significant changes in the output measures. As the calculator is supposed to make "real-time feedback" on changes, we think it is important to avoid long delays when evaluating different diet adjustments. As we have compared higher iterations without significant changes, we would suggest to keep the chosen number of iterations.

The tables and figures were a little confusing. Looking at Table 1, at the estimates for a 20-year-old, and at Figure 1, I would expect the estimates and uncertainty intervals to be the same - as far as I can tell, they are presenting the same data. But they are not - e.g. for female, for a change in legumes from 0 to 200, the table gives values of 2.0 (1.0, 3.2), but the figure reports 2.00 (0.90, 3.10). Most of the values that I checked differ slightly between the table and the figure. Also, in the combined document I was given to review, there is another version of Figure 1 with different estimates and intervals from both table 2 and the first version of figure 1. These inconsistencies are worrying.

> **Response:** Thanks for noting this. Some of the figures and tables did not seem to have been updated when we last modified the time-to-full-effect assumption (and thus some had old values). This has now been fixed. With the last changes that include another change to time-to-full-effect assumption based on comments from another reviewer (10 years as standard, 5, 30, and 50 years as sensitivity analyses), there are some further adjustments in the estimates. In the former draft we presented supplementary file figures with two different ways of calculating uncertainty intervals (one version prersented as uncertainty intervals with our calculations and one version with confidence intervals using crude extracted confidence intervals). We see that this might have been confusing and have opted to present only uncertainty intervals in line with your comments. We have however tried to check more carefully that the tables and figures are correct and consistent.

In fact, since the figure shows the same data as the table (or should, as far as I can tell) I would suggest the table is redundant - the figure is more visually appealing, and includes the actual estimates, so is preferable. The tabular format may be better for the supplement, in order to pack in more information per page, but that doesn't really matter.

> **Response:** We agree that it could make sense to move what was formerly table 2 as a supplementary table.

Another comment on the values reported in the figures - why do all the estimates have zero in the second decimal place? This is too unlikely to be plausible.

> **Response:** Thanks for noting. The calculator through Shiny provided output with one decimal while the Stata plot by default presented two decimals. We have now fixed this so that one decimal is presented in the plots.

In the figures, the uncertainty intervals should not be referred to as "CI" - they are not confidence intervals. "UI" would be more appropriate. Also, is "Effect" the right word for the estimated life years gained?

> **Response:** We see your point. We have now referred to the these as uncertainty intervals should (UI) and written change in life expectancy (LE) as effect label.

In terms of layout, the figures have the TW->FA and TW->OD estimates for each food group together. This is fine, but visually, it would help if each pair of estimates were separated slightly. Alternatively, you could put all the TW->FA estimates together, followed by all the TW->OD estimates, with a gap between the two sections. I guess there are lots of ways these figures could be modified, and finding the optimal layout is not easy.

Response: Thanks for your suggestions. We could not find ways to integrate spacing between each line in the admetan package in Stata (but if you have suggestions to how this is done, we could try to implement that). We checked other sorting strategies, but found these to be less intuitive.

Figure 2 in the paper looks great, except for the fact that it is very hard to tell some of the colours apart. I don't know what could be done about this.

Response: Thanks for that. We have added a note under the figure on lines that are overlapping.

****
